# Development of PVDF Membrane Nanocomposites via Various Functionalization Approaches for Environmental Applications

**DOI:** 10.3390/polym8020032

**Published:** 2016-01-27

**Authors:** Douglas M. Davenport, Minghui Gui, Lindell R. Ormsbee, Dibakar Bhattacharyya

**Affiliations:** 1 Department of Chemical and Materials Engineering, and Center of Membrane Sciences, University of Kentucky, Lexington, KY 40506, USA; dougdvnprt@gmail.com (D.M.D.); guiminghui@gmail.com (M.G.); 2 Department of Civil Engineering, University of Kentucky, Lexington, KY 40506, USA; lindell.ormsbee@uky.edu

**Keywords:** phase inversion, free radical polymerization, polyelectrolyte, water remediation

## Abstract

Membranes are finding wide applications in various fields spanning biological, water, and energy areas. Synthesis of membranes to provide tunable flux, metal sorption, and catalysis has been done through pore functionalization of microfiltration (MF) type membranes with responsive behavior. This methodology provides an opportunity to improve synthetic membrane performance via polymer fabrication and surface modification. By optimizing the polymer coagulation conditions in phase inversion fabrication, spongy polyvinylidene fluoride (PVDF) membranes with high porosity and large internal pore volume were created in lab and full scale. This robust membrane shows a promising mechanical strength as well as high capacity for loading of adsorptive and catalytic materials. By applying surface modification techniques, synthetic membranes with different functionality (carboxyl, amine, and nanoparticle-based) were obtained. These functionalities provide an opportunity to fine-tune the membrane surface properties such as charge and reactivity. The incorporation of stimuli-responsive acrylic polymers (polyacrylic acid or sodium polyacrylate) in membrane pores also results in tunable pore size and ion-exchange capacity. This provides the added benefits of adjustable membrane permeability and metal capture efficiency. The equilibrium and dynamic binding capacity of these functionalized spongy membranes were studied via calcium ion-exchange. Iron/palladium catalytic nanoparticles were immobilized in the polymer matrix in order to perform the challenging degradation of the environmental pollutant trichloroethylene (TCE).

## 1. Introduction

Polymeric membranes have been widely used for environmental applications such as desalination and wastewater treatment [1,2]. Most organic and inorganic compounds can be selectively removed by membranes on the basis of size exclusion, charge repulsion, or a difference in solubility and diffusivity in membrane materials. Compared to inorganic membranes, polymeric membranes are often desirable for their lower cost and greater mechanical flexibility. Cross-linked polymers such as aromatic polyamides are commonly used to provide high performance and highly stable membranes for various desalination applications. The ideal membranes for salt separations have a very thin skin layer for effective separation, and a highly porous supporting layer to enhance membrane stability. A large research effort is focused on improving the skin layer properties of membranes such as anti-fouling, anti-scaling, and high permeability capabilities. However, it is also critical to create membrane substrates with an advanced microstructure and excellent mechanical strength to further enhance their overall separation performance and usefulness [3,4]. The creation of macro-porous adsorptive or reactive membranes will often require different approaches in their fabrication.

Phase inversion is a well-studied immersion precipitation technique for membrane synthesis. Using this common method, polymer is dissolved in an organic solvent and the solution cast upon a flat surface. This thin film is then immersed in a nonsolvent bath medium (in this study water is used) causing the polymer to precipitate from solution and transform to a solid state in a controlled manner [5]. It is possible to control the casting conditions in order to manipulate the thermodynamic process of polymer precipitation and therefore provide control over the resulting membrane structure and morphology [6]. By varying the casting conditions, microfiltration (MF) and ultrafiltration (UF) membranes with various microstructures have been obtained. Among such microstructures, biomimetic sponge-like membranes show very promising applications in bioseparations [7], medical care [8], energy storage [9,10], desalination [11,12], and water remediation [13,14]. Spongy membranes, as defined in this study, are characterized by a thick cross section with an asymmetric, open, and porous interior structure allowing for a maximum possible interior volume available for *in situ* functionalization of membrane pores. With a higher hierarchical order and internal pore volume, spongy membranes usually provide better compressive strength and adsorptive capacity performance than those with conventional structure [15,16,17]. Straight, finger-like pore structure is preferred over spongy and asymmetric pore morphology to minimize internal concentration polarization in some applications such as forward osmosis (FO) type desalination processes [18]. However, the high specific surface area of spongy membranes provides a framework for high-capacity surface and pore functionalization thus increasing effectiveness in various applications.

Functional materials such as polymer ligands or inorganic particles can be easily incorporated into the spongy polymer matrix for enhanced membrane adsorption and selective binding, for instance. A higher loading capacity of functional materials can also be expected for spongy membranes due to the increased pore volume available for functionalization. This could be accomplished using polymer gel with functionality, which is immobilized in spongy membranes via phase inversion or *in-situ* polymerization methods. For example, poly(amidoamine) (PAMAM) functionalized membranes show strong mechanical integrity as well as high chelating capacity for Cu(II) recovery from aqueous solution [19]. In previous studies, cross-linked polyacrylic acid (PAA) has been used as a polyelectrolyte hydrogel inside membrane pores to form an ion-exchange or catalyst membrane platform for metal capture and toxic organic degradation [13]. In these studies, functionalized membranes were prepared at the lab-scale, and reproducibility during scale-up was unknown. Additionally, the dynamic binding capacity and flow-through behavior of large-scale membranes have not been explored. Here, we report the synthesis and optimization of larger-scale spongy PVDF membranes as well as an evaluation of the membrane performance in metal capture and nanoparticle synthesis after surface modification. The goal of this work is to design and develop a high capacity, recyclable, and robust polymeric membrane platform with tunable functionality and selectivity to be efficiently used in low-energy separation or catalytic systems.

## 2. Experimental Section

### 2.1. Materials

All chemicals used in this study were reagent grade without further purification. Acrylic acid, sodium acrylate, sodium borohydride, polyethylenimine (PEI, *M*_w_ = 800), *N,N*-dimethylfomamide (DMF), and lithium chloride were purchased from Sigma-Aldrich (St. Louis, MO, USA). Ferrous chloride tetrahydrate was obtained from Fisher Scientific (Waltham, MA, USA). Ethanol (99.5%), ammonium persulfate (98%), *N,N′*-methylenebisacrylamide (NNMA), 1-(3-Dimethylaminopropyl)-3-ethylcarbodiimide hydrochloride (EDC), and *N*-hydroxysucinimide (NHS) were purchased from Acros Organics (Geel, Belgium). The commercial PVDF powder (Kynar 761) was purchased from Arkema (King of Prussia, PA, USA). Polyvinylpyrrolidone (PVP, *M*_w_ = 40,000) and polyacrylic acid (PAA, *M*_w_ = 50,000, 25% solution) were purchased from Polysciences Inc. (Warrington, PA, USA). The hydrophilized PVDF microfiltration membranes (PVDF400HE and SPVDF, flat sheet) were obtained from Nanostone/Sepro Inc. (Oceanside, CA, USA) PVDF400HE has a thickness of 200 ± 5 µm, including PVDF (75 ± 5 µm) and polyester fabric backing (125 ± 5 µm). The average pore size is 420 nm, and the porosity is 50% ± 5%. Spongy PVDF membrane (SPVDF) has a thickness of 330 ± 5 µm and the porosity of 78% ± 5%, including PVDF (150 ± 5 µm) and polypropylene fabric backing (180 ± 5 µm). All solutions were prepared with Milli-Q ultrapure water (18.2 MΩ·cm at 25 °C). Phosphate Buffered Saline (PBS, 1X) was prepared by dissolving 8 g of NaCl, 0.2 g of KCl, 1.44 g of Na_2_HPO_4_ (10 mM), and 0.24 g of KH_2_PO_4_ in 1 L DI water.

### 2.2. Spongy PVDF Membrane Casting

The spongy PVDF membrane was formed via an optimized phase inversion method based on our previous studies [13]. The casting solution was composed of 81.8 wt % DMF, 15.0 wt % PVDF, 1.5 wt % PVP, and 1.7 wt % LiCl. The casting solution temperature was 35 ± 2 °C, and DI water coagulation bath temperature was 44 ± 2 °C. The membranes were cast on glass plate or commercial supports. A similar formula was used for making the full-scale spongy membranes at Nanostone/Sepro Inc. (Oceanside, CA, USA). For the full-scale, spongy PVDF membranes (40 in wide by approx. 200 ft) were made with similar casting composition using DMF as solvent. A thicker polypropylene commercial backing material was used to minimize curling during the drying process.

### 2.3. Surface Functionalization

PVDF membranes (both lab-scale and full-scale) were functionalized via *in situ* polymerization of either acrylic acid or sodium acrylate (NaAc) in order to immobilize free carboxyl groups within membrane pores. The polymerization solution contains acrylic acid (AA) or sodium acrylate (1.5 M) monomer, initiator ammonium persulfate (1 mol % of monomer), cross-linker NNMA (1 mol % of monomer), and deoxygenated water. Sodium acrylate is the preferred monomer choice because its use will reduce volatile organic compound (VOC) emissions during full-scale manufacturing compared to acrylic acid. A small amount of PAA was also introduced to the polymerization solution to adjust solution viscosity. Membranes were dipped in solution for 30 s and placed with the fabric backing facing upwards in a Buchner funnel. A vacuum was applied at the bottom of funnel to draw the solution into the membrane pores for 5 min. After the excess solution was removed from the external surface via N_2_ purging, membranes were placed between polyethylene sheets, which were sandwiched between glass plates. Cross-linked PAA was formed via heat-initiated polymerization at 90 °C for 35 min. At the conclusion of the reaction time, free radical polymerization was quenched in an ethanol/water mixture (1:1, *v*/*v*), followed by rinsing with DI water. The full-scale functionalization of N-PVDF membrane with PAA was conducted with potassium persulfate as initiator and ethylene glycol as a cross-linker.

In order to functionalize membranes with amine groups, EDC/NHS reagents were used to covalently attach the primary amine groups of PEI to the carboxyl groups of PAA. PAA functionalized PVDF membranes were soaked in EDC/NHS (5 mM) solution for 1 h to activate the carboxyl groups. NaCl (450 mM) and MES buffer (10 mM) were used in this step to maintain the solution pH at 5.5. After rinsing membranes with DI water, membranes were transferred to PEI solution (5 g/L) with PBS buffer (pH 7.4) for 24 h [20].

### 2.4. Iron Nanoparticle in-Situ Synthesis in Membranes

Both zero-valent iron (Fe^0^) and Fe/Pd bimetallic nanoparticles were synthesized via *in-situ* ferrous cation-exchange and borohydride reduction. The carboxyl groups on polymer chains can exchange the iron ions (Fe^2+^) via electrostatic interaction as a precursor for iron nanoparticles. The experimental procedure has been detailed in previous work [21]. It should be noted that, even directly after their synthesis, iron nanoparticles have an iron oxide layer which is less than a few nanometers thick [22]. Our previous work also shows the formation of iron/iron oxide (including ferrihydrite and magnetite) core-shell nanoparticles inside functionalized membranes [21,23]. Other forms of iron oxides can also be obtained depending on the reduction conditions such as pH, precursor state, and oxygen content in water.

### 2.5. Functionalized Membrane Characterization

Electron microscopy was performed using Hitachi S-4300, Zeiss EVO MA 10, and Helios NanoLab 660 DualBeam system (FEI) to study membrane external and internal structure as well as nanoparticle size and morphology. Cross-section images were obtained by focused ion beam (FIB) milling in some cases. Membrane surface charge was monitored by streaming potentials, which were measured by SurPASS Electrokinetic Analyzer (Anton Paar, Graz, Austria). This was done to understand the surface functionality of membranes as the isoelectric points of different surfaces are determined by functional groups attached. Attenuated total reflectance Fourier transform infrared spectroscopy (ATR-FTIR, Varian 7000e) was also used to identify the functional groups on membrane surfaces. Data were collected using 128 scans at a resolution of 4 cm^−1^ in the transmission mode. Mechanical properties of membranes (such as tensile strength) were characterized using an Instron 4442 tensile tester. Membrane samples were cut into dumbbell shape for these tensile tests with ASTM 1708 microtensile die. The concentrations of iron, palladium, and calcium in solutions were analyzed by inductively coupled plasma optical emission spectrometry (ICP-OES, Varian VISTA-PRO, Palo Alto, PA, USA) after acidification with nitric acid (1%). Yttrium chloride (1 mg/L) was used as an internal standard.

### 2.6. Stimuli-Responsive and Ion-Exchange Properties of Functionalized Membranes

The water flux of PAA and PEI functionalized membranes was measured from pH 3 to pH 9 with a stainless steel pressure cell (Sepa ST, GE Osmonics, effective surface area: 13.2 cm^2^). The ion-exchange capacity was calculated as the amount of Ca captured (in meq) per gram of membrane (excluding commercial backing support material weight) for membrane test samples having surface area 13.2 cm^2^. PAA functionalized membranes were preloaded with Na^+^ via cation-exchange in NaCl solution (50 mM, pH = 10), followed by batch soaking or convective flow-through of CaCl_2_ solution (9 mM, pH = 5.3) until adsorption equilibrium was reached.

### 2.7. TCE Dechlorination with SPVDF-PAA-Fe/Pd

**Figure 1:**
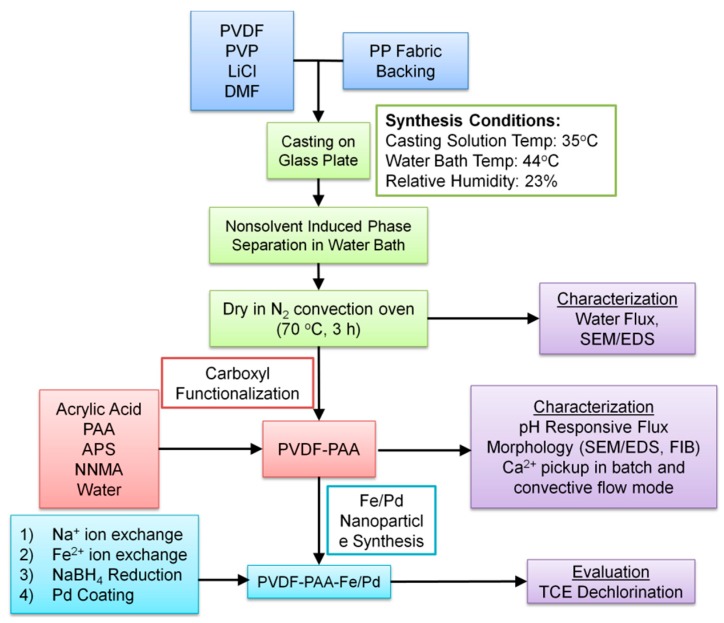
Flowchart detailing spongy membrane synthesis, functionalization, and characterization procedure.

Fe/Pd bimetallic nanoparticles were synthesized in spongy PVDF-PAA membranes via ion-exchange, reduction, and post-coating [24]. These membranes were cut into small pieces before being soaked in TCE solution (0.2 mM, pH = 5.5), followed by mixing at 300 rpm. Membranes and supernatants were extracted separately after the reaction to quantify TCE loss due to membrane adsorption and nanoparticle-catalyzed reduction. A control experiment with only PVDF-PAA membrane (without nanoparticles) and TCE solution was also studied. For TCE analysis, 1, 2-dibromoethane (EDB, internal standard) was premixed with pentane (1:10,000, *v*/*v*), which was used for TCE extraction. Samples were extracted (equi-volume) for 30 min to achieve equilibrium. TCE concentrations were determined by HP 5890 Series II GC-MS with a 60 m × 0.25 mm × 14 μm DB-624 fused silica capillary column (Agilent, Santa Clara, CA, USA). A calibration curve was obtained via plotting the peak area ratios of TCE to EDB with standard concentrations from 0.028 to 0.33 mM (*R*^2^ = 0.997). The concentration of chloride formed during TCE dechlorination was analyzed by DIONEX IC25 ion chromatograph (column: IonPac AS18, 4 × 250 mm) with Na_2_CO_3_/NaHCO_3_ buffer solution as the mobile phase (1 mL/min, 2000 psi). Figure 1 shows a flow chart detailing spongy membrane synthesis, functionalization, and characterization performed in this study.

## 3. Results and Discussion

### 3.1. Membrane Formation and Functionalization

#### 3.1.1. Spongy Membrane Casting

Spongy membranes were cast with a thick cross section and porous, asymmetric morphology in order to obtain a high internal surface area allowing for high functionalization yields. The casting procedure for spongy membrane has been previously reported [13]. It is known that several casting factors such as dope solution composition, polymer molecular weight, coagulation bath medium, casting temperature, and non-solvent additives have a major impact on resulting membrane morphology [5,25,26,27]. These factors were examined and conditions chosen in order to synthesize spongy membranes as defined by a thick and porous cross section with high interior surface area. A significant experimental factor contributing to the desired spongy, asymmetric structure is the membrane casting temperature. An elevated casting temperature favors liquid-liquid demixing prior to polymer crystallization. This results in an asymmetric, porous membrane structure rather than the crystallization-dominated uniform particulate structure experienced at lower casting temperatures [27,28,29]. Therefore, elevating the casting temperature has been chosen as the governing parameter by which to synthesize spongy membranes due to the significant and favorable impact it has on membrane morphology. Thus, the dope solution temperature was increased to 35 °C and coagulation bath to 44 °C, resulting in spongy membrane morphology due to the delayed polymer crystallization during phase inversion.

DMF was selected as the solvent to facilitate the formation of porous and asymmetric membrane structure [27]. A similar solvent such as *N,N*-dimethylacetamide (DMAc), triethylphosphate (TEP) and some mixtures can also be used for spongy membrane casting [6,30]. PVP was dissolved in the dope solution as an additive at a concentration of 10.0 wt % with respect to PVDF (1.5 wt % of total solution). The hydrophilic nature of PVP has multiple effects on membrane morphology and properties. Firstly, it acts as a pore former by enhancing water inflow and liquid-liquid demixing during the phase inversion process, resulting in increased membrane porosity [5]. The second function of PVP is to increase membrane hydrophilicity and aid PAA functionalization during the *in situ* polymerization of aqueous acrylic acid solution. LiCl was also added to the dope solution at a concentration of 11.3 wt % with respect to PVDF (1.7 wt % of total solution). Lithium ions (Li^+^) serve to increase the casting solution viscosity through complexation with negative fluorine dipoles in the PVDF structure. This interaction results in a membrane structure, which is although highly porous, maintains polymer interconnectivity during membrane casting in order to retain the mechanical strength. More importantly, Li^+^ enhances the rate of polymer precipitation during the immersion step due to its high tendency to mix with water (hygroscopic). The formation of more porous structure and larger finger-like macrovoids were reported with increasing the amount of LiCl added to doping solutions [5,31]. The membrane permeability also increased with increasing LiCl concentration while maintaining the same rejection.

**Figure 2:**
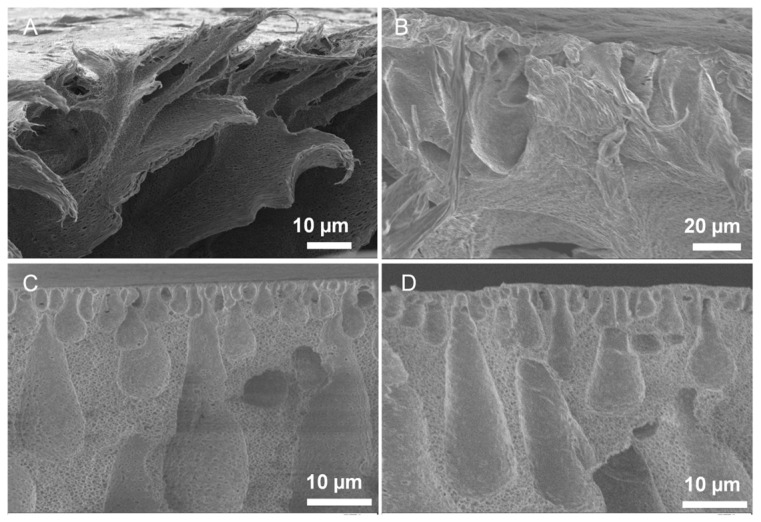
SEM images of non-spongy membrane (N-PVDF) cross-section (**A**); lab-scale spongy membrane L-SPVDF cross-section (**B**); and full-scale spongy membrane N-SPVDF cross-section before polyacrylic acid (PAA) functionalization (**C**); and N-SPVDF after PAA functionalization (**D**).

Table 1 summarizes different spongy membranes tested in this study. Spongy membrane L-SPVDF was synthesized lab-scale while N-SPVDF is a spongy membrane synthesized using full-scale, industrial techniques to create a 40 inch wide membrane sheet. The SEM image of non-spongy membrane (N-PVDF) shows a dense membrane cross-section morphology (Figure 2A). The internal pore structure of L-SPVDF and N-SPVDF membrane are shown in Figure 2B,C, respectively. The asymmetric finger-like structure was formed during the phase inversion process and allows for a high internal pore volume available for further membrane functionalization. As expected, highly porous spongy membranes were obtained with the internal pore size between 0.2 and 0.5 µm.

**Table 1:** Functionalized membrane synthesis conditions and physical properties.

Name	PVDF membrane casting scale	PVDF layer thickness (µm)	Membrane backing material (Thickness in µm)	PAA modification	Mass gain (g PAA/g PVDF × 100%)	Pure water permeability at pH 6 (LMH/Bar)
L-SPVDF-AA ^1^	Lab-scale	175	Polypropylene (175)	Lab-scale	89.5%	13.0
N-SPVDF-AA ^1^	Full-scale	175	Polypropylene (175)	Lab-scale	46.4%	1.7
N-PVDF-AA ^2^	Full-scale	75	Polyester (125)	Full-scale	23.6%	1320
N-PVDF-NaAc ^2^	Full-scale	75	Polyester (125)	Lab-scale	14.6%	137

^1^ Spongy PVDF membrane (SPVDF); ^2^ non-spongy membrane (PVDF).

#### 3.1.2. Surface Modification with Carboxyl Groups and Membrane Characterization

Spongy membranes with highly porous and open internal structure were utilized for PAA functionalization via an *in situ* polymerization of acrylic acid. As shown in Figure 2D, some internal pores with finger-like structure disappeared due to the PAA functionalization. However, the overall pore size and porosity did not change significantly between these finger-like pores. A measure of the PAA loading and membrane functionalization capacity is mass gain. Membrane mass gain is defined as the weight ratio of PAA to PVDF in the membrane as follows:

(1) $$Mass\;gain=\frac{M\prime /A_{m}–M_{0}/A_{m}–M_{f}/A_{f}}{M_{0}/A_{m}–M_{f}/A_{f}}\times 100\%$$

where *M*_0_ and *M*′ are membrane mass before and after functionalization respectively, M_f_ is the fabric mass, and *A*_m_ and *A*_f_ are the membrane and fabric area respectively. Polypropylene fabric backing support weight is disregarded in this calculation as Scanning Electron Microscopy (SEM) and Energy Dispersive Spectroscopy (EDS) results indicate the amount of PAA formed in this domain was not significant. The porous and asymmetric structure of spongy membranes allows for exceptionally high mass gains as high as 89.5% for L-SPVDF-PAA (Table 1). The full scale spongy membranes also show high mass gains on the average of 46.4% for N-SPVDF-PAA. The increased internal pore volume of spongy membranes has provided higher PAA functionalization capacity over the full-scale non-spongy membrane N-PVDF-PAA which had a considerably lower 23.6% mass gain. The spongy structure acted as a supporting scaffold allowing for the enhanced pore modification with a functional polymer. It also shows that the spongy structure allows greater interaction of functional polymer with surrounding media, giving rise to a high dynamic capacity.

**Figure 3:**
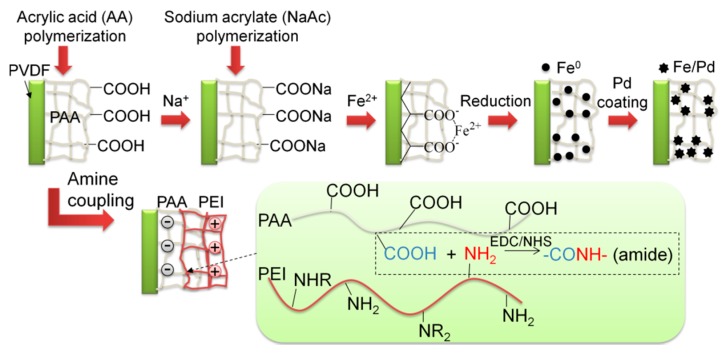
Fe/Pd nanoparticle and amine functionalized membrane synthesis via surface modification.

**Figure 4:**
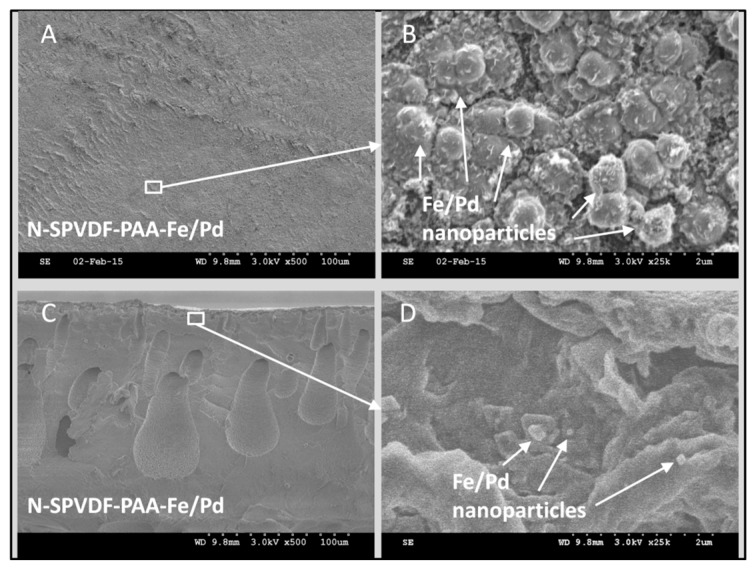
SEM images of N-SPVDF-PAA membrane functionalized with Fe/Pd nanoparticles ranging in size from 0.5 to 3.0 µm. Membrane top surface (**A**,**B**) and cross section (**C**,**D**).

To prove the functionality of spongy membranes to act as high capacity reactive catalyst materials, Fe/Pd nanoparticles were immobilized within PVDF-PAA structure following procedures used in our previous work [23,24]. A schematic is included in Figure 3 summarizing Fe/Pd nanoparticle synthesis as well as amine membrane functionalization. SEM images show Fe/Pd particles were embedded on the top surface (Figure 4A,B) and in the membrane cross section (Figure 4C,D). Particles on the membrane surface are larger in size (1.0–3.0 µm), than those inside (0.5–1.0 µm) due to particle aggregation. Particle size is dependent on the molar ratio of NaBH_4_ to Fe^2+^ during iron reduction, as well as cross linking density. Thus the variation in size indicates the concentration of iron precursor or reducing agent within the membrane pores was different from that in the bulk phase. The use of vacuum to pull functionalization solution through membrane pores during PAA functionalization indicates PAA should be uniformly distributed in membrane pores. However, the addition of PAA to the functionalization solution may not allow for complete passage of monomer solution into the membrane, which resulted in the accumulation of polymer on either surface. Similar phenomenon was reported in dip-coating polymerization processes, and the improvement was obtained in the full-scale process. It is possible to increase the particle loading by repeating the ion exchange process with Fe^2+^ after Fe/Pd has been synthesized [21]. This is because the carboxyl groups in PAA exchange from Fe^2+^ to Na^+^ form during NaBH_4_ reduction, and thus additional Fe/Pd nanoparticles can be loaded in polymer matrix [23].

**Figure 5:**
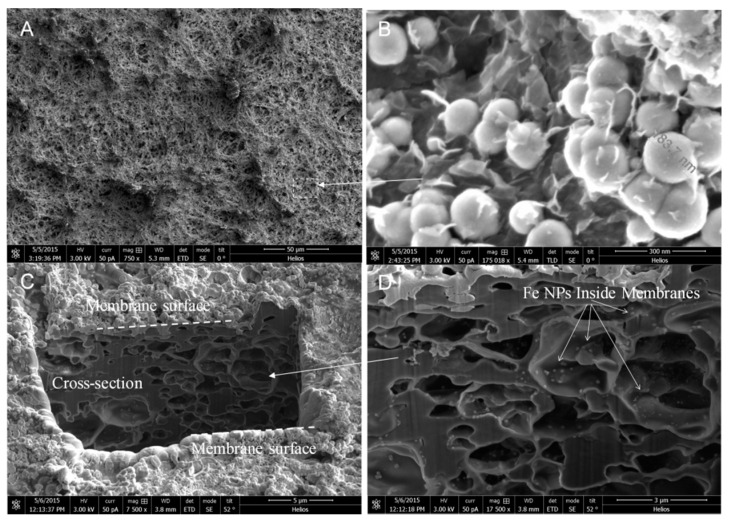
Iron-functionalized N-PVDF-PAA membrane (non-spongy, full-scale) surface and cross-sectional images obtained by FIB. Sodium acrylate (NaAc) was used as the monomer during the polymerization.

In addition to using acrylic acid (AA) for free radical polymerization, sodium acrylate (NaAc) was also polymerized directly to form sodium polyacrylate. Compared to AA, NaAc has a lower vapor pressure and thus generates less volatile organic compounds (VOC) during the manufacturing process. The mass gain for N-SPVDF after polymerization using NaAc was 14.6 wt %. The membrane surface (Figure 5A) and cross-sectional (Figure 5C) morphology were observed using FIB. Using the ion beam milling, the internal porous structure can be clearly seen. A similar external (Figure 5B) and internal (Figure 5D) particle size difference was observed, showing the change of polymer ligand density from the surface to the underlying structure. The size of the iron nanoparticles on the top surface ranges from 100 to 200 nm, while those inside the membrane pores are 30 nm.

#### 3.1.3. Amine Functionalization

EDC/NHS treatment has been widely used to activate carboxyl groups for covalent attachment of primary amine groups (carboxyl-amine conjugation). As shown in the schematic provided in Figure 3, by using excess hyperbranched polyetheneimine (PEI), free amine groups will be available in membrane pores. The reactive amine groups can be used for oxyanion capture and cation rejection directly. They also allow for the functionalized membrane to be used as a robust platform in a variety of applications. All types of functional groups can be further introduced to membrane pores by reacting with amine groups.

**Figure 6:**
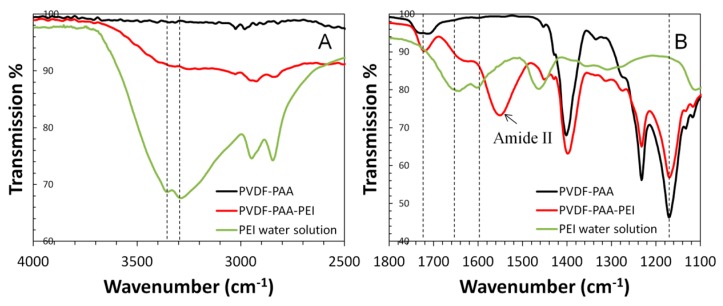
Attenuated total reflectance Fourier transform infrared spectroscopy (ATR-FTIR) of PVDF-PAA membrane (**black** line), PVDF-PAA-PEI membrane (**red** line), and polyethylenimine (PEI) solution (**green** line).

ATR-FTIR spectra of PVDF membrane through surface modification of carboxyl and amine groups are shown in Figure 6. N–H stretch from primary and secondary amines was observed between 3400 and 3250 cm^−1^ while the corresponding N–H bend show between 1650 and 1580 cm^−1^ [20]. The carbonyl (C=O) peak at 1720 cm^−1^ and amide II (C(O)NHR_1_) stretch at 1550 cm^−1^ (combining the N–H bending vibration and C–N stretch) further proved the successful carboxyl-amine conjugation [32]. PVDF-PAA membrane shows a broader peak between 1740 and 1700 cm^−1^ due to the existence of both free carboxylic acids and amides in the cross-linked PAA. PVDF has good stability to the thermal and chemical treatments used in this study, as evidenced by the consistency of C–F stretch at 1182 cm^−1^.

**Figure 7:**
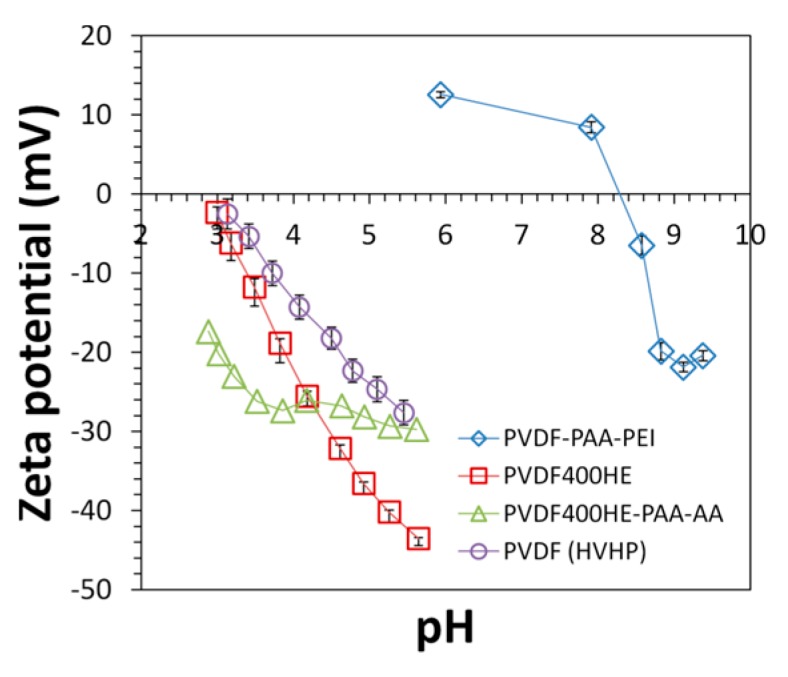
Streaming potentials of PVDF400HE, PVDF400HE-PAA-AA, PVDF400HE-PAA-PEI, and PVDF (HVHP14250) membranes. Electrolyte solution: 0.01 M KCl. PVDF400HE (Nanostone/Sepro) and PVDF400HE-PAA-AA (full-scale) were provided by Nanostone/Sepro. PVDF (HVHP14250) was purchased from EMD Millipore.

The streaming potential results (Figure 7) further confirmed the existence of free primary amine groups in membrane pores. The pristine PVDF membrane has an isoelectric point around pH 3 due to the inherent adsorption of negative ions on its surface. With PAA incorporation in full-scale membrane, there is an obvious phase transition near pH 4, which is near the pKa (4.0–4.9) of carboxyl groups in PAA. The isoelectric point of PVDF-PAA membranes shifted to 8.3 after PEI functionalization, which further indicates the presence of the positive charge of primary amine groups.

### 3.2. Functionalized Membrane Properties

#### 3.2.1. Equilibrium and Dynamic Binding Capacity of Functionalized Spongy Membranes

**Figure 8:**
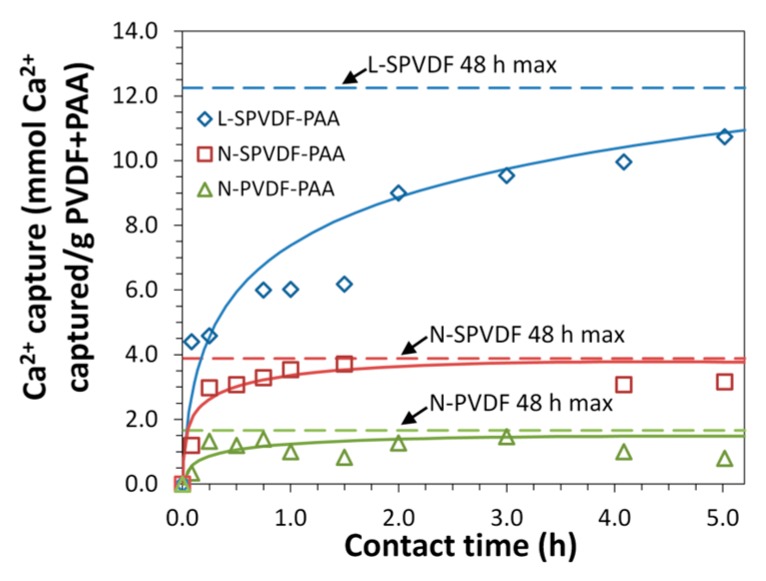
Ion-exchange capacity of functionalized membranes in batch mode. [Ca^2+^] = 1000 ± 20 mg/L in feed solution (pH = 5.8). All membranes were preloaded with Na^+^ in NaCl (pH 11). Results shown are the amount of Ca^2+^ captured per gram of PVDF + PAA (total membrane mass without polypropylene fabric backing). Plotted horizontal asymptotes represent the 48 h maximum Ca^2+^ capture.

**Figure 9:**
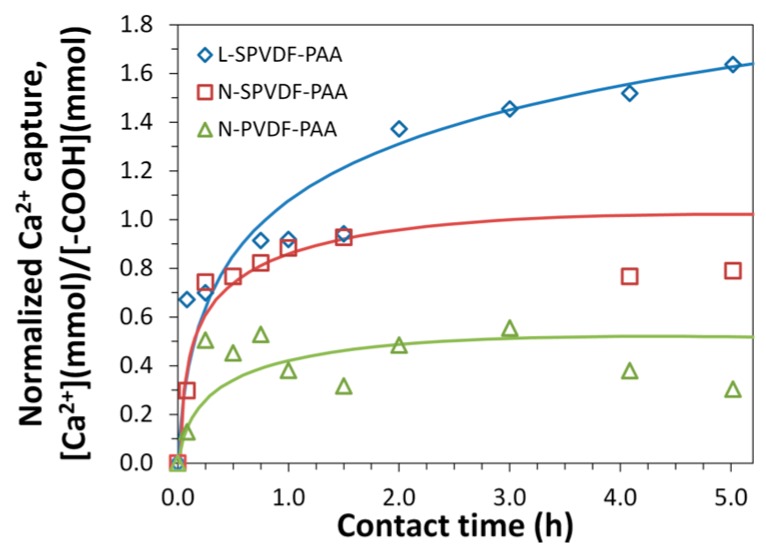
Time course study of Ca^2+^ ion-exchange in batch mode. The feed solution (1000 ± 20 mg/L at pH = 5.8) was freshly prepared using CaCl_2_·2H_2_O. The amount of Ca^2+^ captured was calculated based on the ion-exchange study, while that of –COOH present in the membrane was obtained from the membrane weight gain.

Ion exchange experiments (with Ca^2+^) were performed to quantify the equilibrium and dynamic capacity of PAA functionalized spongy membranes in batch and convective flow conditions. Batch ion-exchange results at pH 5.8 (Figure 8 and Figure 9) show exceptionally high equilibrium binding capacity for L-SPVDF-PAA (24.5 meq/g PVDF+PAA). Support fabric weight is again disregarded due to the absence of PAA in this region. It would be expected that the theoretical calcium capture limit would be a 1:2 molar ratio of Ca^2+^ to –COOH groups present in PAA. However, calcium capture results normalized on the basis of mmol Ca^2+^ capture per mmol –COOH (Figure 9) indicate L-SPVDF-PAA has a 48 h maximum capture of 1.87 Ca^2+^ per carboxyl group. This could be due to the counterion condensation of highly concentrated polyion groups (–COO^−^) in functionalized membranes, which resulted in a high concentration of calcium ions within the polymer domain. Previous studies have shown that counterion condensation has played a significant role in polyion interactions involving PAA polymer chains [33,34]. This can explain the [Ca^2+^]:[–COO^−^] ratio which was seen to be greater than 0.5 for both L-SPVDF-PAA and N-SPVDF-PAA. An increased calcium capture capacity was observed for spongy membranes over non-spongy N-PVDF-PAA. This indicates that the open internal pore volume of spongy membranes allows for greater availability of carboxyl groups to contact Ca^2+^ rich solution. This increased availability of functional groups in spongy membranes resulted in a greater capture of calcium via ionic interaction with carboxyl groups and also through counterion condensation. Therefore, the spongy membranes have exceptionally high equilibrium capacity.

The experiments were done at a constant temperature of 21 °C. In the above figures, the curves passing through the points show the data trend (not a model fit). The L-SPVDF (see Table 1) has the highest % functionalization of PAA in the pores and thus one would expect significant conformation change through Ca sorption, and thus further enhancing cation capture through “counterion condensation”.

**Figure 10:**
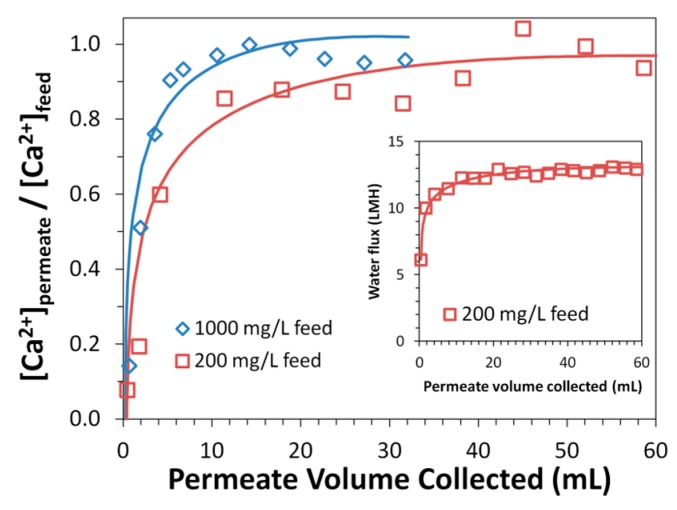
Normalized permeate concentration change during Ca^2+^ pickup with N-SPVDF-PAA functionalized in convective flow mode. Inset shows the change in water flux for 200 mg/L CaCl_2_ solution throughout experimental run. Feed solutions were passed through membranes using a dead-end filtration cell at 10.3 bar.

In addition to its higher ion-exchange capacity, the spongy membrane also shows a higher rate of calcium capture than the non-spongy membrane. Spongy membranes show a high dynamic binding capacity through the rapid capture of calcium in batch experiments (Figure 8). Calcium ion-exchange experiments were also performed in a convective flow-through system for N-SPVDF-PAA to further examine the rate of calcium capture. Feed solutions with Ca^2+^ concentrations of 1000 mg/L and 200 mg/L (pH 6.1 and 5.7 respectively) were pressurized to 150 psi (10.2 bar) in a dead end filtration cell. The breakthrough curve for each experiment (Figure 10) rapidly reached its maxima indicating membrane saturation with Ca^2+^. This rapid calcium capture is again attributed to the open and spongy structure of the N-SPVDF-PAA membrane, which gives rise to this high dynamic capacity. Macrovoids present in the membrane might limit the effective utilization of functionalized regions, and thus the calcium capture efficiency of the membrane can be further improved by reducing the extent of macrovoids. The control of macrovoids can be obtained through polymer concentration, type of solvent used, and kinetics of phase separation. It is also interesting to note that water flux increased as calcium adsorbed in the membrane. This is due to the complexation of ionized carboxyl groups with Ca^2+^ captured in the membrane. In its ionized form, cross-linked PAA chains have a swollen conformation within membrane pores, thus decreasing flux, as –COO^−^ groups repel from other negatively charged groups on the polymer chain. The complexation of PAA with Ca^2+^ has the same function as H^+^ to increase membrane flux by neutralizing the charge of carboxylic acid functional groups.

**Figure 11:**
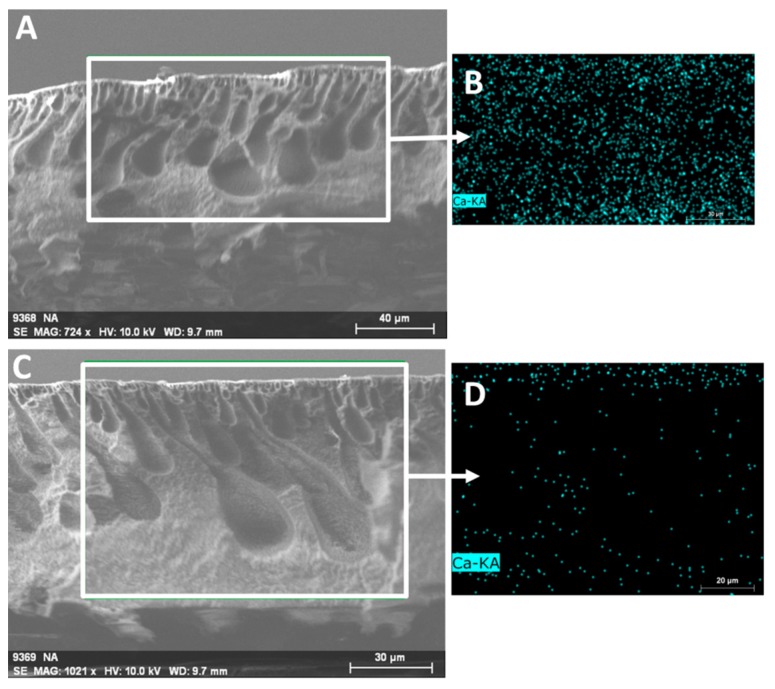
SEM imaging and EDS mapping (Ca) of N-SPVDF-PAA membrane cross section (PVDF layer only). (**A**) and (**B**) are N-SPVDF-PAA after Ca^2+^ ion-exchange (200 mg/L feed solution) in batch mode, while (**C**) and (**D**) are pristine N-SPVDF-PAA directly following PAA functionalization.

To prove that membranes were uniformly functionalized with PAA, EDS analysis was performed on samples of N-SPVDF-PAA before and after calcium ion-exchange experiments. Figure 11A,B show an N-SPVDF-PAA sample cross section following 24 h reaction time in a batch setup with 200 mg/L Ca^2+^ at pH 5.6. Figure 11C,D show the cross-section of a pristine membrane functionalized with PAA which underwent no interaction with a calcium solution. The presence of trace amounts of calcium is visible in the pristine N-SPVDF-PAA sample; however, it is clear there is a much greater presence of Ca^2+^ in the sample following batch Ca^2+^ ion-exchange. It should be noted that there is no increased concentration of Ca^2+^ near the surface or any other area of the membrane. Instead, Ca^2+^ is uniformly distributed throughout the membrane cross section. This indicates that the open and spongy structure allows for highly efficient ion interactions with PAA functional groups throughout the membrane structure.

#### 3.2.2. Stimuli-Responsive Pores

**Figure 12:**
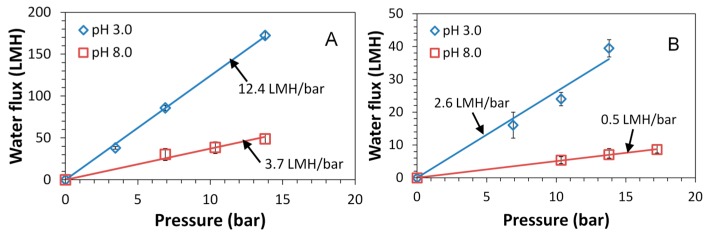
pH responsive flux behavior of spongy membranes L-SPVDF-PAA (**A**) and N-SPVDF-PAA (**B**). Flux tests were performed in a dead end filtration cell and pH is adjusted using 0.1 M HCl and NaOH.

**Figure 13:**
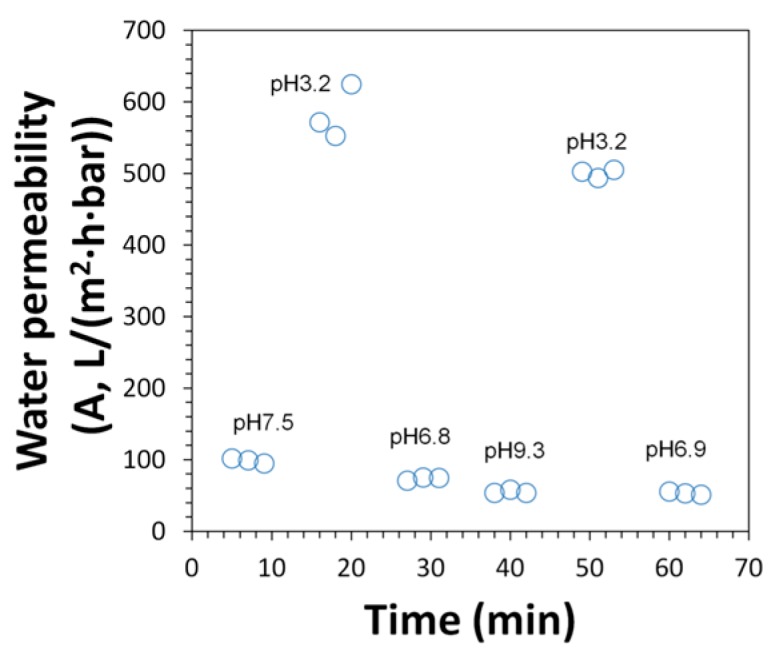
Water flux of N-PVDF-NaAc membrane functionalized by sodium acrylate (NaAc) polymerization at different pH. Membrane weight gain: 14.6%.

Previous studies have shown the significance of pH responsive flux behavior due to PAA presence in membrane pores [13,35,36]. The effective pore size of PAA functionalized membrane can be controlled by the protonation and deprotonation of carboxyl groups (–COOH). This transformation is sensitive to water pH, and results in the swelling and deswelling of PAA gels. The pH responsive water flux is again shown in Figure 12 as a proof of PAA functionalization within membrane pores. Spongy membranes L-SPVDF-PAA and N-SPVDF-PAA show a flux ratio of 3.4 and 5.2 respectively for pH variation from pH 3.0 to pH 8.0. Non-spongy membranes (N-PVDF-NaAc) functionalized by sodium polyacrylate also show similar responsive behavior of permeability as a result of changes in pH (Figure 13).

#### 3.2.3. Mechanical Strength

There are a great number of cellular biomaterials existing in nature which are mechanically efficient due to their unique structure and design. By implementation of similar structure, synthetic materials with enhanced mechanical strength may be developed [37]. These biologically inspired materials may help us better understand the novel functional mechanisms found in nature.

However, tensile tests show that there was a reduction in the mechanical strength for spongy membranes. The tensile modulus of non-spongy membranes was twice as high as spongy membranes (Table 2). A similar drop (1.4 times) was seen on tensile yield strain for N-SPVDF. Although the spongy materials have a higher hierarchical order and interconnected three-dimensional structure [15], the formation of macrovoids during phase inversion reduces the mechanical strength of membranes significantly [38]. The membrane thickness, the thickness ratio of PVDF layer and supporting layer, and the non-woven backing tensile strength could also determine the performance of the fabricated membrane. Additionally, the supporting layer surface energy or hydrophobicity could influence performance. After PAA pore functionalization, both membranes show enhanced tensile modulus and yield strain. When the internal pores were filled by cross-linked PAA hydrogel, the interaction between scaffold PVDF units became stronger. Additionally, acrylic polymers are widely used as coating binders, so the PAA gel might play a role as an adhesive-like interlayer, which connects the internal pores and uniformly distributes the tensile force. During the tensile tests, all membranes show irreversible stretching. Previous studies show the changes of crystalline structure of PVDF during the stretching process [39]. Therefore, a relatively low pressure (up to 18 bar) was applied in membrane permeability tests to maintain the membrane and PAA hydrogel structure. The compressed gel or crushed polymer network would have lower permeability and weaken pH responsive behavior. This was observed in our repeated water permeability test of sodium polyacrylate functionalized membranes (Figure 13).

**Table 2:** Mechanical properties of PVDF and functionalized membranes.

	Tensile modulus (MPa)	Tensile yield strain (MPa)
N-SPVDF ^1^	156 ± 14	18 ± 1
N-SPVDF-AA	273 ± 9	24 ± 4
N-PVDF ^2^	310 ± 22	25 ± 1
N-PVDF-AA	543 ± 45	29 ± 4

^1^ Spongy PVDF membrane (N-SPVDF); ^2^ non-spongy membrane (N-PVDF).

### 3.3. TCE Dechlorination with Fe/Pd Functionalized Membranes

**Figure 14:**
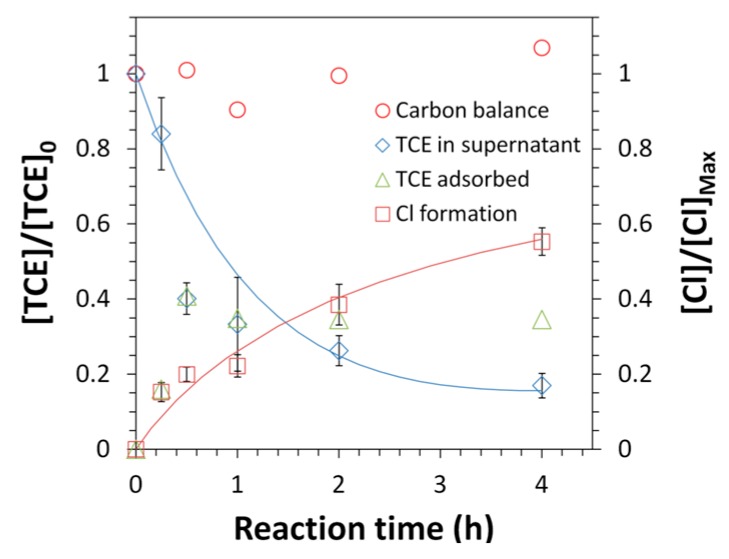
Trichloroethylene (TCE) dechlorination and chloride formation by Fe/Pd functionalized N-SPVDF membranes. TCE in supernatant analyzed by extraction of supernatant while TCE adsorbed analyzed by extraction of membrane after reaction with TCE. Cl^−^ formation results from the analysis of supernatant for chloride ions using ion exchange chromatography. [Fe]_0_ = 0.58 ± 0.02 g/L, [Pd]_0_ = 1.25 ± 0.25 wt % of [Fe]_0_. [TCE]_0_ = 0.28 mM, *V* = 20 mL. The maximum chloride concentration is 0.83 mM (complete dechlorination).

The dechlorination of TCE in deionized water was tested with N-PVDF-PAA-Fe/Pd membranes and the results were reported in Figure 14. The decreasing TCE concentration in solution was measured by GC-MS while the formation of chloride as a product of TCE dechlorination was measured using ion exchange chromatography. This functionalized spongy membrane shows a significant production of Cl^−^ up to 0.46 mM within 4 h with 0.28 mM TCE as feed. Three moles of Cl^−^ per mole of TCE should form assuming complete dechlorination of TCE and no loss of TCE to the atmosphere. Therefore, this result equates to a chloride production of 55% of the theoretical maximum. By extracting the membranes after the TCE reaction, it can be seen that spongy membranes show a high partitioning of TCE in the membrane phase. It is seen that about 40% of TCE was adsorbed in 15 min, and so the TCE introduced to the solution was not only degraded but adsorbed by the membrane at a high rate. A control experiment was performed with an identical membrane, N-SPVDF-PAA, in the absence of catalytic Fe/Pd nanoparticles. As expected, no chloride formation was observed in the control run despite consistent adsorption of TCE. The spongy membrane provides a porous support structure for functionalization with PAA and Fe/Pd nanoparticles leading to high and rapid dechlorination of organic compounds such as TCE. In a convective flow setup, the TCE dechlorination rate is expected to be enhanced due to the absence of diffusion resistance from the bulk solution phase to the membrane pore region. The catalytic reactivity of the membrane can be further improved by reducing the number of macrovoids to ensure efficient utilization of functionalized regions. The behavior of the PVDF-PAA polymer matrix to adsorb high quantities of TCE indicates spongy membranes could also be potentially used as super adsorbent materials. In doing so, these spongy membranes functionalized with Fe/Pd nanoparticles offer promise to be utilized in the environmental remediation of toxic metals and/or chlorinated organic compounds. These are important environmental separations not easily done by other methods.

## 4. Conclusions

By controlling the rate of liquid demixing and polymer crystallization during phase inversion, sponge-like PVDF membranes with highly porous structure were synthesized in both lab and full-scale (40 in wide). With the immobilization of pH responsive PAA hydrogel via *in-situ* polymerization of acrylic acid or sodium acrylate, functionalized membranes with tunable pore size and high binding capacity were obtained. Spongy membranes achieved a Ca^2+^ capture equilibrium capacity as high as 24.5 meq/g PVDF+PAA, compared to 3.2 meq/g for non-spongy membranes. The dynamic binding capacity was also studied by Ca^2+^ capture under convective flow. Spongy membranes have a maximum capture efficiency of 1.87 Ca^2+^ per carboxyl group, which was more than four times higher than that for non-spongy membranes. The ion-exchange equilibrium was quickly reached, and water flux increased due to the shrinking of the hydrogel after Ca^2+^ chelation with PAA. The high hierarchical order of the sponge-like structure and acrylic polymer pore functionalization both contributed to the enhanced mechanical strength of spongy membranes as evidenced by tensile strength tests. These responsive membranes have also been functionalized with Fe/Pd bimetallic nanoparticles and have been used for the degradation of TCE. This offers promise for these materials to be used for environmental remediation applications such as toxic metal capture and chlorinated organic compound degradation.
